# Quantification and factors associated with HIV-related stigma among persons living with HIV/AIDS on antiretroviral therapy at the HIV-day care unit of the Bamenda Regional Hospital, North West Region of Cameroon

**DOI:** 10.1186/s12992-018-0374-5

**Published:** 2018-06-05

**Authors:** Atem Bethel Ajong, Philip Nana Njotang, Ngholapeh Emmanuel Nghoniji, Marie José Essi, Martin Ndinakie Yakum, Valirie Ndip Agbor, Bruno Kenfack

**Affiliations:** 1 Kekem District Hospital, Kekem town, West Region Cameroon; 2 Department of Obstetrics and Gynaecology, Faculty of Medicine and Biomedical Sciences, University of Yaoundé I, Yaoundé, Cameroon; 3 Obstetrics and Gynaecology unit, Higher Institute of Health Technologies, Yaoundé Central Hospital, Yaoundé, Cameroon; 4 Bamenda Regional Hospital, Bamenda, North West region Cameroon; 5 Department of Public Health, Faculty of Medicine and Biomedical Sciences, University of Yaoundé I, Yaoundé, Cameroon; 6 Meilleur Accès aux Soins de Santé, Yaoundé, Cameroon; 7 Ibal sub-Divisional Hospital, Oku, North-west Region Cameroon; 8 Department of Biomedical Sciences, University of Dschang, Dschang, Cameroon

**Keywords:** Human immunodeficiency virus, Acquired immunodeficiency syndrome, Antiretroviral therapy, Stigma, Cameroon

## Abstract

**Background:**

The Human Immunodeficiency Virus /Acquired Immune Deficiency Syndrome (HIV/AIDS) is not just a medical problem but its social impact is increasingly affecting its effective management. The fear of HIV-stigma constitutes a major barrier to HIV testing, prevention, uptake and adherence to antiretroviral therapy (ART). We aimed to quantify HIV-related stigma, and identify the factors associated with high HIV-related stigma among persons living with HIV and AIDS (PLHIVA) and on ART.

**Methods:**

A hospital-based cross sectional analytic survey targeting PLHIVA on ART at the HIV-day care unit of the Bamenda Regional Hospital of Cameroon was conducted from February to April 2016. A total of 308 eligible and willing participants were consecutively included in the survey. Data were collected using a pretested questionnaire designed from the Berger HIV stigma scale and analyzed using Epi info 3.5.4.

**Results:**

The mean age of the 308 participants was 40.1±10.2 years. The mean overall HIV/AIDS related stigma score was 88.3 ± 18.80 which corresponds to a moderate level of stigma according to the Berger stigma scale. Further analysis revealed that most participants suffered from moderate forms of the different subtypes of stigma including: personalized (49.8%), disclosure (66.4%), negative self-image (50.0%) and public attitude (52.1%) stigmatization. It was estimated that 62.7% (95% confidence interval [CI] = 57.8–68.9%) of the participants lived with high levels of HIV-related stigma. After controlling for gender, religion, age and occupation, level of education below tertiary (Adjusted Odds Ratio [AOR] = 0.70 [95% CI = 0.44–0.91]; *p* = 0.036) and a duration from diagnosis below 5 years (AOR = 1.74 [95% CI = 1.01–3.00]; *p* = 0.046) were significantly associated with high HIV-related stigma.

**Conclusion:**

About three out of every five PLHIVA receiving ART in Bamenda Regional Hospital still experience high levels of HIV-related stigma. This occurs more frequently in participants with low educational status, and who may have known their HIV status for less than 5 years. Anti-HIV-stigma programs in the North West Region need strengthening with intensified psychosocial follow-up of newly diagnosed cases.

**Electronic supplementary material:**

The online version of this article (10.1186/s12992-018-0374-5) contains supplementary material, which is available to authorized users.

## Background

The Human Immunodeficiency Virus /Acquired Immune Deficiency Syndrome (HIV/AIDS) remains one of the most stigmatizing pandemics worldwide [1]. The fight against HIV/AIDS remains one of the major points in the sustainable development agenda [2]. Though great efforts have been implemented at different levels to limit the spread of HIV/AIDS, the incidence of HIV in the developing world and more precisely Sub-Saharan Africa (SSA) is still relatively high [3].

By the end of 2015, an estimated 36.7 million people were living with HIV/AIDS worldwide, with over two-thirds residing in sub-Saharan Africa, which includes Cameroon [4]. Despite the recently reported drop in the prevalence of HIV in Cameroon from 5.5 to 4.3% over the last decade [5], it still remains amongst the highest in West and Central Africa [6].

Tackling AIDS-related stigma and discrimination is crucial in the effective prevention of HIV/AIDS, the care of PLHIVA (People Living with HIV and AIDS) and goes a long way to significantly help in containing and managing this pandemic [1, 7–10]. HIV-related discrimination is not only a human right violation according to the United Nations General Assembly Special Session on HIV/AIDS, but problems of HIV-related stigmatization and discrimination need to be properly addressed to successfully meet public health goals [11, 12].

The prevalence of HIV-related stigma varies with the setting and method of evaluation. Even though it has no standardized method of evaluation, HIV-related stigma is reported to be highly prevalent among PLHIVA [11–15]. At every level of the HIV/AIDS management ladder (the prevention, care and treatment), stigma and discrimination has seriously impeded its success [12]. Unlike other chronic conditions such as diabetes, hypertension and chronic liver disease, HIV testing and status disclosure are seriously limited by stigma and discrimination [13–19]. Negative predictors of HIV-related stigma include, but are not limited to: a good knowledge on HIV/AIDS, past participation in HIV anti-stigma campaigns, low level of education, and religion [19]. HIV-related stigma and discrimination is a major barrier limiting participation in prevention of mother to child transmission programs, HIV treatment, and adherence to ART [19]. HIV-related stigma is therefore a serious barrier towards the successful attainment of the ambitious triple 90 goal (90% diagnosed, 90% on treatment and 90% with suppressed viral load) of the Joint United Nations programme on HIV/AIDS (UNAIDS) by the year 2020 [20].

In Cameroon, despite diverse efforts put in place by the Cameroon health system to combat HIV-related stigma and discrimination, HIV-related stigma and discrimination remains a significant obstacle to the fight against HIV/AIDS and the adherence to HIV/AIDS related counseling [5]. To the best of our knowledge, no study has been done to quantify HIV-related stigma and identify its associated factors in Cameroon. A quantification of HIV-related stigma will not only serve as a baseline for future comparison, but will help to evaluate the anti-stigma strategies integrated in the management guidelines. Identifying factors associated with HIV-related stigma might help redesign or reinforce these interventions. Therefore, we sought to measure HIV-related stigma, and identify factors associated with high HIV-related stigma among PLHIVA on antiretroviral therapy (ART) at the HIV-day care unit of the Bamenda Regional Hospital.

## Methods

A hospital-based cross sectional analytic survey was conducted from February to April 2016. All eligible and consenting persons living with HIV/AIDS (PLHIVA) on ART at the HIV-day care unit of the Bamenda Regional hospital were targeted and recruited. The HIV-day care unit of the Bamenda Regional Hospital is a reference HIV treatment unit in the North-west Region of Cameroon, and receives patients from both the rural and urban areas of the Region. We excluded PLHIVA who were mentally incapacitated, below 15 years of age and patients who arrived critically ill and unable to respond to the questionnaire. ART-naïve patients were not included because according to the Test and Treat recommendation, any patient not yet on treatment should just have been awaiting confirmation of his or her status. All eligible and consenting participants who visited the treatment unit during the study period were consecutively included in the survey (*n* = 308 PLHIVA).

With the aim of limiting surveyor induced stigmatization and discrimination, the principal investigator undertook a two-months internship at the HIV-day care unit of the Bamenda Regional Hospital, and recruited colleagues as interviewers. Training sessions were organized during which interviewers were trained on the consent process and data collection procedures. When the protocol, the questionnaire and informed consent form were completed, the data collection tools were pretested on a sample 15 PLHIVA in a treatment centre in Yaoundé (Centre region of Cameroon) and validated after analysis by a team of experts. Berger HIV Stigma Scale validated by Feyissa et al. in a resource-limited setting (Ethiopia) [21] and Jeyaseelan et al. in India [22], contains four subscales (domains): Enacted Stigma, Disclosure Concerns (a form of anticipated stigma), Negative Self-Image (e.g. internalized stigma), and Concern with Public Attitudes towards HIV (e.g., anticipated stigma) [21].

This culturally validated, HIV-Stigma scale has been used with good results particularly in clinical settings to identify patients in need of psycho-emotional support and assess post-intervention changes in stigma in many settings [23, 24]. It should be noted that onto the elements of the Berger scale was added sociodemographic data and some information that allowed the identification of factors associated with HIV-related stigma. The questionnaire was paper-based and is attached as (Additional file 1). Interviewers were of both sexes and were allowed to interview willing participants irrespective of gender. During data collection, the interviewer identified eligible participants, and after thorough explanation of the information notice form of the study, obtained informed consent from the participant or legal guardian. The data were collected face to face by interviewers in private rooms within the facility (one participant at a time) who asked questions and took down the responses of each participant. Participants received no remunerations for taking part in the survey.

### Data analysis

Data from validated questionnaires were entered into a predesigned data entry sheet. After cleaning, the data were analyzed using the statistical software Epi-Info version 3.5.4. Categorical variables are reported as proportions with their corresponding 95% confidence intervals (CI), while means were calculated for continuous variables. HIV-related stigma level was categorized into “high” and “low”. Participants with “high” HIV-related stigma were those for which the Berger stigma scale classified them into at least a moderate level of stigma (that is, participants with a mean score value greater than or equal to 80). The strength of association between the selected covariates (level of education, estimated monthly income, marital status, number of years from detection of HIV status, place of residence) and the stigma level (high or low) was determined by calculating the Odds Ratio (OR) and their 95% CI. A multivariable logistic regression analysis was used to identify factors associated with high HIV-related stigma (with gender, religion, age, and occupation considered as potential confounders; solely based on literature). The threshold of statistical significance was set at a *p*-value less than 0.05.

## Results

A total of 331 PLHIVA were contacted for the study and 23 refused to participate giving a final sample size of 308 PLHIVA and a non-response rate of 6.9%. The mean age of the 308 participants was 40.1±10.2 years with a mean age of 43.9 ± 8.4 years and 38.1 ± 10.6 years among men and women respectively (*p* < 0.001). The population was dominated by women (65.3%). About 2/5th (40.9%) of the participants were married, 35.1% single and 54.9% had acquired at least secondary education. Christians were most represented (95.8%) with the rest Muslims. More than 2/3th (69.9%) of the participants reported estimated monthly revenue of less than 50000FCFA (80.75 United States dollars).

Table 1 presents the ranges and means of the different domains of stigma experienced by the participants. The overall mean HIV-related stigma score was 88.3 ± 18.80 (with a possible range of 40–160) which corresponds to a moderate level of stigma according to the Berger stigma scale. The mean HIV-related stigma score was 87.65 ± 18.62 and 88.64 ± 18.89 in men and women respectively (*p*-value = 0.6608).

**Table 1:** Ranges and means of the different domains of stigma experienced by the participants

Stigma domain(lowest to highest score)	Minimum score recorded	Maximum score recorded	Mean score ± SD in males	Mean score ± SD in females	*p*-value	Mean***±*** SD^a^(male and female)
Personalized (18–72)	18	65	40.06 ± 10.51	39.93 ± 9.75	0.9195	39.98±10.00
Disclosure (10–40)	12	34	22.87 ± 4.65	23.67 ± 4.80	0.1596	23.39±4.76
Negative self- image (13–52)	14	48	26.96 ± 6.15	27.58 ± 6.85	0.4235	27.38±6.61
Public attitude (20–80)	20	73	45.09 ± 11.44	44.99 ± 11.05	0.9382	45.03±11.17
HIV stigma overall (40–160)	44	138	87.65 ± 18.62	88.64 ± 18.89	0.6608	88.3±18.80

^a^Standard Deviation

Table 2 shows the frequency distribution of respondents according to HIV-stigma score level. Overall, 37.3, 58.4% (180/308) and 4.2% experienced mild, moderate and severe HIV-related stigma respectively. Following a subgroup analysis, irrespective of gender, it was noted that most patients suffered from moderate forms of personalized (49.8%), disclosure (66.4%), negative self-image (50.0%) and public attitude (52.1%) stigmatization. The proportion with high level of stigmatization among the population was 62.7% (95% CI = 57.8–68.9%).

**Table 2:** Frequency distribution of respondents on the HIV stigma score level

Stigma type	Score level	Score value	Frequency (percentage) in males	Frequency (percentage) In females	Frequency (percentage)
Personalized	Mild	18–36	45(42.5%)	82(41.2%)	127(41.6%)
	Moderate	37–54	49(46.2%)	103(51.8%)	152(49.8%)
	Severe	55–72	12(11.3%)	14(7.0%)	26(8.5%)
Disclosure	Mild	10–20	29(27.4%)	53(26.4%)	82(26.7%)
	Moderate	21–30	75(70.8%)	129(64.2%)	204(66.4%)
	Severe	31–40	2(1.9%)	19 (9.5%)	21(6.8%)
Negative self- image	Mild	13–26	51(47.7%)	90(44.8%)	141(45.8%)
	Moderate	27–39	54(50.5%)	100(49.8%)	154(50.0%)
	Severe	39–40	2(1.9%)	11(5.5%)	13(4.2%)
Public attitude	Mild	20–40	42(39.6%)	74(37.2%)	116(38.0%)
	Moderate	41–60	53(50.0%)	106(53.3%)	159(52.1%)
	Severe	61–80	11(10.4%)	19(9.5%)	30(9.8%)
HIV stigma overall	Mild	40–80	38(36.2%)	77(36.7%)	115 (37.3%)
	Moderate	81–120	63(60.0%)	117(58.8%)	180(58.4%)
	Severe	121–160	4(3.8%)	9(4.5%)	13(4.2%)

Table 3 presents the factors associated with high HIV-related stigma following bivariable and multivariable logistic regression analysis. When controlled for gender, religion, age, and occupation; level of education below tertiary (Adjusted Odd Ratio [AOR] = 0.70 [95% CI 0.44–0.91]; *p* = 0.036) and duration from diagnosis below 5 years (AOR = 1.74[1.01–3.00]; *p* = 0.046) were independently associated with high HIV-related stigma. There was no statistically significant association between high HIV-related stigma and place of residence (rural/urban), monthly revenue and marital status.

**Table 3:** Factors associated with high HIV/AIDS related stigma

Factors	Univariable analysis			Multivariable analysis^a^		
	OR	95% CI	p-value	AOR	95%ACI	p-value
Level of education above secondary (Y/ N)	0.69*	0.43–0.89	0.032	0.70	0.44–0.91	0.036*
Number of year since diagnosis of HIV(+) Less than 5 years(Y/N)	1.74*	1.02–2.98	0.042	1.74	1.01–3.00	0.046*
Urban residence(Y/N)	1.41	0.82–2.42	0.210	1.35	0.78–2.35	0.287
Monthly revenue less than 50.000FCFA(Y/N)	0.97	0.60–1.62	0.956	0.84	0.47–1.51	0.567
In union (Y/N)	0.84	0.53–1.35	0.479	0.84	0.51–7.53	0.489

Where Y/N=Yes/No, *OR* odds ratio *CI* confidence interval, *AOR* adjusted odds ratio, *ACI* adjusted confidence interval, *statistically significant (*p* ≤ 0.05)

^a^multivariable analysis was done with with gender, religion, age, and occupation considered possible confounders

## Discussion

The mean overall HIV/AIDS related stigma score was 88.3 ± 18.80 which corresponds to moderate level of stigma according the Berger stigma scale. In addition, most participants suffered from moderate forms of the different subtypes of stigma including: personalized (49.8%), disclosure (66.4%), negative self-image (50.0%) and public attitude (52.1%) stigmatization. Over 60% of the participants lived with high levels of HIV-related stigma. A level of education below tertiary and duration from diagnosis below 5 years were significantly associated with high HIV-related stigma.

The sex ratio reported in this study is similar to that reported among PLHIVA in Africa with a female preponderance [6, 25]. In addition, national demographic and health survey report in 2011 shows that in Cameroon, the ratio is about two women infected with HIV for every one man [5]. This is in correlation with data on the prevalence of HIV/AIDS in Cameroon and more precisely in the North West Region of Cameroon where surveys have shown that more women are infected with HIV/AIDS than men [5]. The higher prevalence in women simply brings to light vulnerability of women to the HIV/AIDS infection, mainly due to biological factors, which are usually favoured by socio-behavioural practices and socioeconomic differences [26]. The overall mean HIV-related stigma score was 88.3 ± 18.80 (with a possible range of 40–160) which corresponds to a moderate level of stigma according the Berger stigma scale. Even though women seem more likely to be infected by the virus (sex ratio of about 2:1), the mean HIV-related stigma score was 87.65 ± 18.62 and 88.64 ± 18.89 in men and women respectively (with no statistically significant difference. *p*-value = 0.6608). This means that on average, most clients in the HIV-treatment unit experienced moderate HIV-related stigma and that both genders were similarly affected. This is true as each of the estimated means of the stigma sub-types was found to fall at least into the moderate stigma category. HIV-related stigma has been reported high and highlighted as a barrier to HIV/AIDS management by a series of African surveys [5, 25, 27, 28]. This study indicates that HIV-related stigma is still very common among PLHIVA taking treatment from the Bamenda Regional Hospital. A study carried out in Ethiopia reported similarly high levels of perceived HIV-related stigma mean score values [29].

Following a subgroup analysis, irrespective of gender, it was noted that most patients suffered from moderate forms of personalized (49.8%), disclosure (66.4%), negative self-image (50.0%) and public attitude (52.1%) stigmatization. The proportion with “high” level of stigmatization among the population was 62.7% (95% CI = 57.8–68.9%). This is indicative of the high rate of HIV-related stigma perceived by these participants. Multiple studies in the African context have reported similarly high rates of HIV-related stigma among HIV positive patients [29–31]. These high rates of stigma could be associated with multiple pitfalls in the effective management of HIV/AIDS in Africa. According to investigators of the 2011 national DHS in Cameroon, 46 and 58% of women and men respectively had never been screened for HIV [5]. High rates of HIV-related stigma have been associated with decreasing level of HIV voluntary testing [14, 32–34], status disclosure [25, 32], adherence to ART [32, 35], educative programs on HIV transmission and increased probabilities of maintaining the transmission chain [32]. If patients received at the treatment centres are this stigmatized, it gives us an idea of the level of stigmatisation and the effect the effect that this can have among patients lost to follow-up and others in the community.

Indeed, up to 66.4% of the participants had moderate problems with disclosure. This form of HIV-related stigma could be associated with very low rates of HIV sero-status disclosure and consequently maintenance of the transmission chain [7]. This high level of HIV-related stigma can seriously impede on the attainment of the ambitious triple 90 HIV management goals by the UNAIDS, set for 2020 [20].

In Cameroon, anti-HIV-stigma interventions have been adopted at the national, local (Health units and the community) and individual level. Interventions at the national level include, but are not limited to: legislative protection of the rights of PLHIVA, facilitation of access to family planning services, emergency obstetric care and ART, and organisation of trainings to reduce stigma and discrimination in schools and among health care providers. Interventions at the community level include among others: improving the knowledge of community dwellers on the transmission of HIV/AIDS, correcting perceptions through educative talks, and encouraging leaders to create a climate of tolerance with no prejudice. The health care provider can reduce HIV-related stigma by avoiding stigmatizing statements, adoption of attitudes void of judgement, preserving and respecting patient-provider confidentiality, and adopting universal standard precautions for all patients regardless of their HIV-status.

The fight against this pandemic in Africa and more precisely in Cameroon requires much more focus in redesigning educative programs for both HIV positive and negative individuals in order to break this underground barrier. HIV-related stigma maintains the epidemic underground and remains one of the major explanations why people do not wish to know their HIV status [7, 36].

After controlling for gender, religion, age and occupation, a level of education below tertiary and a duration from diagnosis below 5 years were significantly associated with high HIV-related stigma. The findings in this study suggest that PLHIVA with at least a tertiary education are less likely to be victims of high HIV-related stigma compared to their counterparts with lower level of education. A high level of education is associated with a better understanding of HIV/AIDS [5] and consequently, reduced disease-related stigma [14, 16, 36, 37]. A facility-based cross-sectional Ethiopian study in 2015 reported a high level of education to be significantly associated with reduced HIV-related stigma among PLHIVA [29]. It is clear that a high level of education and awareness among the population on HIV/AIDS could limit the amount of stigma and discrimination associated with the AIDS pandemic [28]. Research findings suggest that improvingliteracy rates and a focus on correcting incorrect conceptions on HIV/AIDS might go a long way in fighting HIV-related stigma [14, 31].

Clients who had been diagnosed within the last 5 years were more likely to be victims of high levels of stigma compared to those who had lived with their diagnosis for more than 5 years. Some level of “normalization” of the HIV status and stigma reduction has been reported in surveys by provision of ART to PLHIVA [32, 38]. This finding is coherent with that of Fido et al., where a decreasing trend of HIV-related stigma was associated with increasing number of years on ART [29]. Similarly, Tzemis et al. reported a decreasing trend of HIV-related stigma with the years of living with HIV/AIDS and years on antiretroviral therapy [37]. Intensified psychological counselling and emotional support in the early days following HIV diagnosis can help PLHIVA to gradually accept and live with their status.

Our study found no significant associations between place of residence (urban/rural), estimated monthly revenue, and marital status and high level of HIV-related stigma. Contrasting findings were reported by Tzemis et al. concerning these variables [39]. This could be explained by a relatively lower number of patients per variable modality in the current study (compared to the study of Tzemis et al.), with consequential lower statistical power. In addition, the sociocultural and financial status of the participants in the current study and that of Tzemis et al. are different.

The findings herein should however be interpreted with care. The cross-sectional design of this study only allows development of hypotheses and cannot establish any cause-effect relationships. In addition, no randomization or power calculation was conducted. Also, recruitment of participants was essentially convenient as data collection was dependent on the study duration. Even though our interviewers were well trained, the responses of some participants could be made socially desirable rather than expressing their actual feelings, thereby underestimating the true burden of stigmatization in PLHIVA. Although the Berger HIV stigma scale has a few limitations, it has been reported as an effective measure of stigma levels especially for future comparisons. In addition, the findings from a single treatment unit in Cameroon cannot fully paint the picture of the reality in Cameroon and sub-Saharan Africa. Also, our study enrolled only patients on ART and systematically excluded those who are not taking treatment. This can possibly lead to an underestimation of the level of HIV/AIDS-related stigma in the whole population since we think that patients who are loss to follow-up would be possibly more stigmatized than the study participants. However our findings were consistent with results of studies carried out in Africa and SSA and can be used as a baseline for future estimates and comparison.

## Conclusion

About 3 out of every 5 PLHIV receiving ART in the HIV-day care unit of the Bamenda Regional Hospital still experience high levels of HIV-related stigma. This occurs more frequently in participants with low educational status, and who may have known their HIV status for less than 5 years. The overall mean HIV-related stigma score among these patients remains high. Anti-HIV-stigma programs in the North West Region need strengthening with intensified psychosocial follow-up of newly diagnosed cases. Improving literacy rates may also help in reducing HIV-related stigma. Large scale studies that quantitatively measure HIV-related stigma in more treatment centers are required; so as to establish a clearer picture of HIV-related stigma in Cameroon.

## Additional file

Additional file 1: Questionnaire for the evaluation of HIV-related stigma among PLHIVA and associated factors. (DOCX 32 kb)

## Abbreviations

AIDS: Acquired immune deficiency syndrome
AOR: Adjusted odds ratio
ART: Antiretroviral therapy
CI: Confidence interval
DHS: Demographic and health survey
HIV: The human immunodeficiency virus
PLHIVA: People living with HIV and AIDS
SSA: Sub-Saharan Africa
UNAIDS: Joint United Nations Programme on HIV/AIDS
