# Supplementary material for: Quantification and factors associated with HIV-related stigma among persons living with HIV/AIDS on antiretroviral therapy at the HIV-day care unit of the Bamenda Regional Hospital, North West Region of Cameroon
Source: Global Health. 2018 Jun 5;14:56. doi: 10.1186/s12992-018-0374-5 (PMC5987427; doi:10.1186/s12992-018-0374-5)
Supplement: Supplementary file 1 — Questionnaire for the evaluation of HIV-related stigma among PLHIVA and associated factors. (DOCX 32 kb) [file 12992_2018_374_MOESM1_ESM.docx]

**QUESTIONAIRE**

**Date………………………………………**

**Name of interviewer……………………………………………..**

**Code of participant: …/…/…/…/…..**

**A) Socio-demographic data**

1) Age: 15-25 25-35
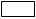
 35-45
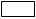
 45-55
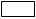
 55+
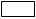


2) Sex: male
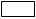
 female
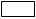


3) Marital status:

Single
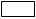
 married
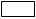
 divorce
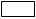
 widow/widower
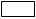


4) Level of education:

Never schooled
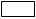
 Primary
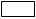
 secondary
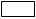
 higher
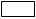


Koranic
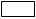


5) Religion: Catholic
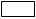
 Protestant
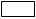
 Muslim
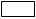
 Atheist
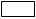
 Animist
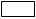


6) Occupation: Skilled job
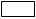
 unskilled
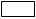


7) Cultural area. Grass field
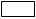
 Forest
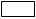
 savannah
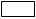
 Coast
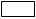


Sahel
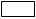
 Pigmy
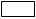


8) Number of years since when you were diagnosed HIV positive……………………

9) What’s your estimated monthly revenue?

Less than 50000
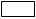
 50000-100000
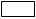
 100000-200000
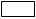


200000-250000
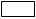
 above 250000
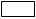


10) HIV exposure : heterosexual
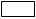
 gay/lesbian
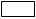
 drug users
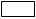
 others
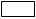


**B) HIV/AIDS stigmatization (The Berger HIV stigma scale)**

For each item, circle your answer: Strongly disagree (SD), disagree (D), agree (A), or strongly agree (SA).

1. In many areas of my life, no one knows that I have HIV...................................................................................................... SD D A SA

2. I feel guilty because I have HIV.................................................... SD D A SA

3. People's attitudes about HIV make me feel worse about myself....................................................................................................SD D A SA

4. Telling someone I have HIV is risky................................................ SD D A SA

5. People with HIV lose their jobs when their employers find out......................................................................................................... SD D A SA

6. I work hard to keep my HIV a secret...............................................SD D A SA

7. I feel I am not as good a person as others because I have HIV.......................................................................................................SD D A SA

8. I never feel ashamed of having HIV............................................... SD D A SA

9. People with HIV are treated like outcasts ......................................SD D A SA

10. Most people believe that a person who has HIV is dirty...................................................................................................... SD D A SA

11. It is easier to avoid new friendships than worry about telling someone that I have HIV..................................................................................................... SD D A SA

12. Having HIV makes me feel unclean.............................................. SD D A SA

13. Since learning I have HIV, I feel set apart and isolated from the rest of the world.................................................................................................... SD D A SA

14. Most people think that a person with HIV is disgusting ............................................................................................................. SD D A SA

15. Having HIV makes me feel that I'm a bad person......................... SD D A SA

16. Most people with HIV are rejected when others find out………..SD D A SA

17. I am very careful who I tell that I have HIV .................................SD D A SA

18. Some people who know I have HIV have grown more distant ............................................................................................................... SD D A SA

19. Since learning I have HIV, I worry about people discriminating against me ..............................................................................................................SD D A SA

20. Most people are uncomfortable around someone with HIV ..............................................................................................................SD D A SA

21. I never feel the need to hide the fact that I have HIV ...............................................................................................................SD D A SA

22. I worry that people may judge me when they learn I have HIV ...............................................................................................................SD D A SA

23. Having HIV in my body is disgusting to me................................... SD D A SA

24. I have been hurt by how people reacted to learning I have HIV .............................................................................................................. SD D A SA

25. I worry that people who know I have HIV will tell others .............................................................................................................. SD D A SA

26. I regret having told some people that I have HIV .......................... SD D A SA

27. As a rule, telling others that I have HIV has been a mistake ............................................................................................................... SD D A SA

28. Some people avoid touching me once they know I have HIV ............................................................................................................... SD D A SA

29. People I care about stopped calling after learning I have HIV ................................................................................................................ SD D A SA

30. People have told me that getting HIV is what I deserve for how I lived my life ................................................................................................................ SD D A SA

31. Some people close to me are afraid others will reject them if it becomes known that I have HIV .................................................................................................. SD D A SA

32. People don't want me around their children once they know I have HIV ….............................................................................................................. SD D A SA

33. People have physically backed away from me when they learn I have HIV …………………………………............................................................... SD D A SA

34. Some people act as though it's my fault I have HIV ........................... SD D A SA

35. I have stopped socializing with some people because of their reactions to my having HIV.............................................................................................................. SD D A SA

36. I have lost friends by telling them I have HIV...................................... SD D A SA

37. I have told people close to me to keep the fact that I have HIV a secret …………………………………................................................................. SD D A SA

38. People who know I have HIV tend to ignore my good points ……………………….............................................................................. SD D A SA

39. People seem afraid of me once they learn I have HIV …………………….................................................................................. SD D A SA

40. When people learn you have HIV, they look for flaws in your character..................................................................................................... SD D A SA

SCORING for the Berger HIV Stigma Scale and Subscales

1) Items are scored as follows: strongly disagree = 1 disagrees = 2 agree = 3 strongly agree = 4. If a subject selects a response in between two options (e.g.: between SD and D), a numerical value midway between the two options would be used (e.g.: 1.5).

2) Two items are reverse-scored: items 8 and 21.

3) After reversing these two items, each scale or subscale’s score is calculated by simply adding up the raw values of the items belonging to that scale or subscale. Subscale designations appear in small print in the far right margin of the instrument; it may be desirable to cover or delete those numbers before reproducing the instrument for administration to subjects. Sixteen items belong to more than one subscale, reflecting the inter-correlations of the factors on which the subscales are based.

4) The range of possible scores depends on the number of items in the scale. For the total HIV Stigma Scale, scores can range from 40 to 160 [1 x 40 items to 4 x 40 items]. For the personalized stigma subscale, scores can range from 18 to 72. For the disclosure subscale, scores can range from 10 to 40. For the negative self-image subscale, scores can range from 13 to 52. For the public attitudes subscale, scores can range from 20 to 80.
